# BetaAlign: a deep learning approach for multiple sequence alignment

**DOI:** 10.1093/bioinformatics/btaf009

**Published:** 2025-01-08

**Authors:** Edo Dotan, Elya Wygoda, Noa Ecker, Michael Alburquerque, Oren Avram, Yonatan Belinkov, Tal Pupko

**Affiliations:** The Shmunis School of Biomedicine and Cancer Research, George S. Wise Faculty of Life Sciences, Tel Aviv University, Tel Aviv 69978, Israel; The Henry and Marilyn Taub Faculty of Computer Science, Technion—Israel Institute of Technology, Haifa 3200003, Israel; The Shmunis School of Biomedicine and Cancer Research, George S. Wise Faculty of Life Sciences, Tel Aviv University, Tel Aviv 69978, Israel; The Shmunis School of Biomedicine and Cancer Research, George S. Wise Faculty of Life Sciences, Tel Aviv University, Tel Aviv 69978, Israel; The Shmunis School of Biomedicine and Cancer Research, George S. Wise Faculty of Life Sciences, Tel Aviv University, Tel Aviv 69978, Israel; The Department of Computer Science, University of California Los Angeles, Los Angeles, CA 90095, United States; The Henry and Marilyn Taub Faculty of Computer Science, Technion—Israel Institute of Technology, Haifa 3200003, Israel; The Shmunis School of Biomedicine and Cancer Research, George S. Wise Faculty of Life Sciences, Tel Aviv University, Tel Aviv 69978, Israel

## Abstract

**Motivation:**

Multiple sequence alignments (MSAs) are extensively used in biology, from phylogenetic reconstruction to structure and function prediction. Here, we suggest an out-of-the-box approach for the inference of MSAs, which relies on algorithms developed for processing natural languages. We show that our artificial intelligence (AI)-based methodology can be trained to align sequences by processing alignments that are generated via simulations, and thus different aligners can be easily generated for datasets with specific evolutionary dynamics attributes. We expect that natural language processing (NLP) solutions will replace or augment classic solutions for computing alignments, and more generally, challenging inference tasks in phylogenomics.

**Results:**

The MSA problem is a fundamental pillar in bioinformatics, comparative genomics, and phylogenetics. Here, we characterize and improve BetaAlign, the first deep learning aligner, which substantially deviates from conventional algorithms of alignment computation. BetaAlign draws on NLP techniques and trains transformers to map a set of unaligned biological sequences to an MSA. We show that our approach is highly accurate, comparable and sometimes better than state-of-the-art alignment tools. We characterize the performance of BetaAlign and the effect of various aspects on accuracy; for example, the size of the training data, the effect of different transformer architectures, and the effect of learning on a subspace of indel-model parameters (subspace learning). We also introduce a new technique that leads to improved performance compared to our previous approach. Our findings further uncover the potential of NLP-based methods for sequence alignment, highlighting that AI-based algorithms can substantially challenge classic approaches in phylogenomics and bioinformatics.

**Availability and implementation:**

Datasets used in this work are available on HuggingFace (Wolf et al. Transformers: state-of-the-art natural language processing. In: *Proceedings of the 2020 Conference on Empirical Methods in Natural Language Processing: System Demonstrations*. p.38–45. 2020) at: https://huggingface.co/dotan1111. Source code is available at: https://github.com/idotan286/SimulateAlignments.

## 1 Introduction

The Needleman–Wunsch algorithm was the first to use dynamic programming to efficiently find the best global scoring alignment between two sequences (Needleman and Wunsch 1970). The inference of a multiple sequence alignment (MSA) was later shown to be an NP-complete problem (Wang and Jiang 1994), making the inference task impractical for a large set of sequences. To overcome this hurdle, popular MSA algorithms, such as MAFFT (Katoh and Standley 2013) and PRANK (Löytynoja 2014), use heuristics to reduce the search space and consequently, the running time.

There is extensive knowledge regarding the variability of the evolutionary process among different datasets and lineages. For example, amino acid replacement matrices vary between proteins encoded in the nuclear genome, the mitochondria, and plastids (Pesole *et al.* 1999). Indel dynamics also highly vary between datasets and among different phylogenetic groups (Wolf *et al.* 2007, Ajawatanawong and Baldauf 2013, Loewenthal *et al.* 2021). Furthermore, site-specific evolutionary rates vary along the analyzed sequence. For example, amino acid sites that are exposed to the solvent tend to have higher evolutionary rates compared to buried sites (Wang *et al.* 2008). Alignment algorithms using default configurations implicitly assume that the evolutionary dynamics do not substantially vary among different datasets and within a single dataset. The general inability of MSA inference algorithms to automatically tune their scoring scheme to the specific dataset being analyzed is a shortcoming of present alignment programs. The “one matrix fits all biological datasets” and “one matrix fits all regions within a dataset” assumptions implicitly employed by most current methodologies raise fundamental questions about the correctness of alignments produced by such methods. Although it is possible to modify gap-penalty parameters in some alignment programs, these programs do not provide means to automatically tune the parameters to specific datasets or regions within a dataset, and hence, by and large, all users employ the default settings.

Alignment algorithms are typically assessed by empirical alignment regions, but these regions are not comprehensive enough to cover the entire range of alignment challenges. These regions are often calculated manually, so their reliability as a “gold standard” is uncertain (Iantorno *et al.* 2014). Differences exist between empirical and simulated datasets, e.g., the latter may not account for evolutionary scenarios such as micro-rearrangements (Walker *et al.* 2021). Thus, when alignment programs are tested with simulated alignments, the results may differ from empirical benchmark outcomes (Chang *et al.* 2014).

One of the key concepts in learning algorithms, in general, and in deep learning algorithms in particular, is the ability to learn from previously annotated data, i.e., to generalize from previous observations to unseen cases. For the task of alignment inference, a deep learning algorithm should learn from “true” alignments (e.g., simulated sequences for which the correct alignment is known) and apply the obtained knowledge to align novel sequences. In this work, we aimed to harness natural language processing (NLP) learning algorithms to the task of aligning sequences; thus, to better capture the evolutionary dynamics of biological sequences.

Here, we present an improvement for our previously developed BetaAlign approach (Dotan *et al.* 2023), in which instead of computing a single alignment, we infer multiple alternative alignments and return the one that maximizes the certainty. To further characterize BetaAlign, we conducted the following analyses: (i) evaluating the effect of training time and size; (ii) evaluating the effect of transfer learning; (iii) measuring the performance as a function of the evolutionary dynamics that generated the sequences; and (iv) comparing different transformer architectures. We also introduce the term subspace learning to describe training on a subspace of the indel parameters and investigate its utility for BetaAlign. Lastly, we show that the benefit of our approach is also transferable, that is, the embedding obtained by the model could serve as meaningful features for accurate inference in other learning tasks such as inferring sequence length prediction of ancestral sequences. Table 1 describes the main differences between the previous and current work. For completeness, we start by describing the algorithm.

**Table 1.** The different topics discussed in this research compared to the previous version of BetaAlign.

Topic	What is new in this work
Algorithm: increasing the accuracy by generating alternative alignments for the same set of unaligned sequences and selecting the best one	We changed our alignment methodology. In the new algorithm, we calculate multiple alternative alignments and return the alignment that maximizes the certainty. Thus, all the results in the current manuscript are new, as they are computed with the novel alignment algorithm
Analysis: the effect of training time and size	We investigated the effect of the training phase on BetaAlign’s loss and performance
Analysis: the effect of indel model parameters on BetaAlign performance	We investigated the effect of indel parameters on BetaAlign’s performance
Analysis: subspace learning	We introduce the term subspace learning to describe training on a subspace of the indel parameters. We investigate how subspace learning affects BetaAlign’s performance
Algorithm: embedding extraction for downstream tasks	We introduced a new approach to gather meaningful representations of unaligned and aligned sequences and evaluate its performance
Analysis: transfer learning	We investigated the effect of transfer learning on BetaAlign’s performance
Analysis: architecture comparisons	We investigated the effect of different transformer architectures
Algorithm: handling invalid alignments and long sequences	These issues were explained in our previous paper and are hence only shortly described here

## 2 Materials and methods

### 2.1 New approach

2.1.1 Outline

**Figure 1.**
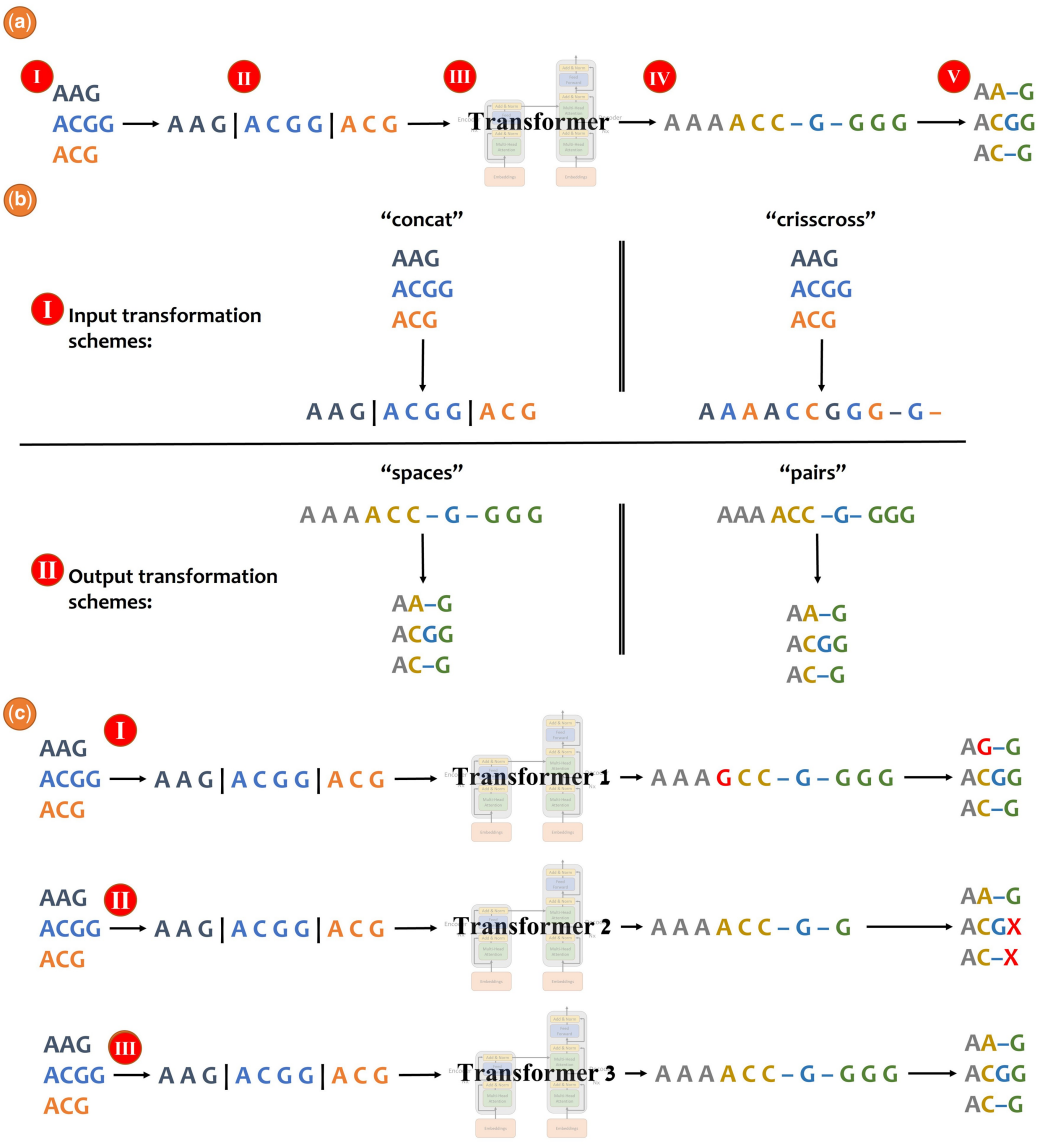
**Example of aligning three sequences with BetaAlign,** (a): (I) Consider the unaligned sequences “AAG”, “ACGG” and “ACG”; (II) The unaligned sequences are concatenated to a single sentence with a special character “|” between each original sequence; (III) The trained model processes the single input sentence and generates the single output sentence; (IV) The processed output is structured such that the first three nucleotides represent the first column, the next three nucleotides represent the second column, and so on; (V) The output is converted into an MSA. (b) An illustration of the different input (I) and output (II) transformation schemes. (c) Example of handling invalid alignments. When aligning the same sequences, BetaAlign first transformer may mistakenly mutated the character “A” to “G” (I); A different transformer resulted in a different output, may generate a shorter sequence in which the last two characters are missing (II); The third transformer provided a valid alignment as output and can be used as the output of BetalAlign (III).

Typically, sequence-to-sequence NLP tasks involve a single sentence (or text) as both input and output, e.g., translating from one language to another or changing a sentence from active to passive (Sutskever *et al.* 2014, Bahdanau *et al.* 2016, Shalumov and Haskey 2023). The learning phase of the algorithm is to map a single input sentence to a single output sentence. When we aim to apply sequence-to-sequence models to the problem of alignment, we are faced with a challenge: the input to the alignment task is several “sentences”, each corresponding to an unaligned sequence. Similarly, the output is a set of related sentences, each corresponding to a row in the resulting alignment. The first task in the BetaAlign algorithm is to transform the set of unaligned sequences to a single “sentence”. Such input- transformation can be done, e.g., by concatenating all the unaligned sequences, adding a special character (we use the pipe character, “|”) to indicate the boundaries between the sequences (Fig. 1). For training the algorithm, we also need to provide target sentences. Thus, we also need an output-transformation step, in which we convert resulting alignments to a single target sentence. In BetaAlign, we use the “*spaces*” representation (Fig. 1). The above representations allow providing a sequence-to-sequence model with a large set of examples of valid source and target sentences, which are used for model training. The models that we use rely on the transformer architecture (Vaswani *et al.* 2017). Once trained, the optimized transformer can process new unseen examples, in our case, it can transform (unseen) unaligned sequences to an alignment.

There are several aspects that need to be addressed to fully describe the BetaAlign algorithm and how its performance was evaluated. These include, for example, the generation of training and test data, the transformer architecture and how it was trained, the handling of long sequences and how the generation of invalid alignments was prevented. We aim to provide a more general description as part of the New approach section, while technical details are provided in Materials and Methods.

### 2.1.2 Generation of training and test data

For both training and testing the performance of BetaAlign, many sets (data points) of unaligned sequences and their corresponding “true alignment” were needed. These data points were generated using simulations. Specifically, we use SpartaABC (Loewenthal *et al.* 2021), which allows different length distributions for insertions and deletions. For example, the initial testing and training for the pairwise alignment problem were achieved by generating millions of pairs of two unaligned sequences and their corresponding alignments for the training and testing data. The indel rates, their type (insertion or deletion), and their length distribution were sampled from specific ranges. We note that we do not assume equal rates of insertions and deletions, nor equal length distributions for the two types of events (this is mainly important when simulating along a tree rather than when simulating pairwise alignments).

### 2.1.3 Transformer architecture

Transformers are currently the working horse of NLP and other AI domains. A transformer is a deep learning model designed to handle discrete sequential data. The transformer used in our work is composed of an encoder and a decoder. The encoder embeds each input sequence (and in our case, the source sequence representing multiple unaligned sequences) into a sequence of high-dimensional vector representation. In this vector space, two related sequences should be closer to each other than two less related sequences. This projection from the sequence space to the high-dimensional space, i.e., the embedding process, is not fixed, but rather is learned as part of the training process. Next, the decoder receives those representations and the last generated token and predicts the next token (a token in natural languages is the building block of a sentence, in our case, each token is either a base pair or an amino acid). The encoder and decoder are neural networks with multiple sequential layers, each containing numerous neurons. These neurons act as linear functions with tunable parameters that are adjusted during training. To handle complex data, each layer of the transformer also incorporates non-linear functions (without non-linear functions, the model acts as a function composition of linear only functions which results in a linear function). Although each layer could theoretically attend to all previously computed embeddings (i.e., the input to the layer), previous research has shown that focusing on a specific subset of tokens yields better results. This approach, known as attention, is a foundational aspect of the transformer model (Vaswani *et al.* 2017). To create the initialized set of the embeddings, the discrete data (in our case, the DNA and the amino acid sequences) are converted via the tokenizer into a set of ids (Dotan *et al.* 2024), which are then converted to a numerical representation via the embeddings matrix. Transformers may vary in architecture, number of layers, and size. These features are the tunable architectural hyperparameters. When training a transformer, one can also vary the learning hyperparameters, e.g., the parameter “max tokens” determines how much input to process before the model parameters are updated. We have tested several transformer architectures and parameters, implemented using the Fairseq library (Ott *et al.* 2019).

### 2.1.4 Transfer learning and subspace learning

The input and output patterns of the analyzed sequences vary as a function of their number, e.g., the number of pipe characters in the “*concat*” representation. We thus optimized a different transformer for each number of sequences. To this end, when optimizing the transformer for, say, five sequences, we start the parameter optimization step from the set of optimal parameters obtained for the previous transformer that was trained on four sequences, a technique called transfer learning (Tan *et al.* 2018, Avram *et al.* 2024).

We also use transfer learning in order to train a transformer on subregions of the parameter space, i.e., subspace learning (see Section 2.9). For example, we can train a general pairwise alignment transformer as described above and then train a different transformer only for alignments with a high ratio of indels to substitutions. In essence, this allows training several transformers, specialized for subregions of the parameter space.

### 2.1.5 Handling invalid alignments

Transformers have no inherent mechanism that restricts them to generate valid alignments. Thus, in some cases, a trained transformer may produce invalid outputs. For example, when aligning sequences, each output sequence, including gap characters, should be of the same length (Fig. 1c). To this end, we trained several different transformers, which differ from each other with respect to their tunable hyperparameters, on the same training dataset (see Section 2.3). If a transformer provided an invalid alignment, we provided the output of an alternative transformer.

### 2.1.6 Handling long sequences

The transformers that we have utilized were designed to process text of natural languages and not biological sequences. As such, they are limited to processing sentences with up to 1024 tokens. When aligning biological sequences, the input and output sentences often exceed this length threshold. Due to memory and run-time constraints, increasing the threshold is infeasible. To overcome this challenge, we introduced a “segmentation” methodology, in which we align segments of the alignments, which are later concatenated to form the entire MSA. This procedure is achieved by training dedicated transformers for this task (Dotan *et al.* 2023).

### 2.1.7 Considering alternative input and output transformation schemes

The transformer architectures we harnessed for the task of aligning sequences are sequence-to-sequence models. One of the key components of our proposed alignment approach is to transpose the multiple input sequences into a single sentence that can be processed by the transformer. Input transformation converts the unaligned sequences into the “input sentence” of the transformer, while output transformation converts the “output sentence” of the transformer into an MSA.

There are various transformation schemes available for converting unaligned sequences into a single sentence. In Fig. 1a, we present the “*concat*” representation: the unaligned sequences are concatenated with a special character “|”. The vocabulary, which encompasses the entire set of possible tokens, of this scheme is {“A”, “C”, “G”, “T” and “|”} for the nucleotide sequences. We used the “*spaces*” representation for output transformation, in which each of the amino acids or nucleotides is considered a separate token. The vocabulary of this scheme for DNA sequences is {“A”, “C”, “G”, “T” and “–”}.

However, alternative transformation schemes for the source sequences can be considered. We previously considered the “*crisscross*” scheme, the tokens of the unaligned sequences are interleaved (Dotan *et al.* 2023). That is, the first token represents the first character from the first unaligned sequence, the second token represents the first token of the second unaligned sequence, and so on. The vocabulary of this scheme is {“A”, “C”, “G”, “T” and “–”} for the nucleotide sequences. Of note, the gap character is used to fill the gaps if the sequences are of different lengths (Fig. 1b). Similarly, alternative transformation schemes for generating the output sentence are possible.

In the “pairs” scheme, each token represents the entire column. The vocabulary of this scheme depends on the number of unaligned sequences, for instance, when aligning three DNA sequences, the vocabulary size is 124 tokens: {“AAA”, “AAC”, “AAG”, “AAT”, “AA–”, …, “TTG”, and “TTT”}. Of note, the token “– – –” (three gap characters) is invalid as such column cannot exist.

It is important to remember that the transformation schemes are external to the transformer itself. Each transformation methodology creates a different mapping from unaligned sequences to an MSA, which requires training the transformer on these representations. Different considerations come into play when selecting the appropriate scheme (Dotan *et al.* 2023). In the “*pairs*” scheme, the output sequence length is the number of columns, while in the “*spaces*” the length is the number of nucleotides. Because length is a limiting factor when using current transformer architectures, using the “*pairs*” scheme may be advantageous. However, the “*pairs*” scheme restricts the use of transfer learning (see below). When transitioning from pairwise alignment to aligning three sequences, the vocabulary would change (from 24 tokens to 124 tokens) and in general, the number of possible tokens exponentially increases as a function of the number of unaligned sequences. In our previous work, we observed that the “*concat*” and “*spaces*” representations (shown in Fig. 1a) performed best (Dotan *et al.* 2023). Thus, all the experiments in this work are done with these representations for the input sequences and output MSA, respectively.

### 2.1.8 Increasing the accuracy by generating alternative alignments for the same set of unaligned sequences and selecting the best one

We present a method for generating multiple alternative MSAs from the same input data. This is done by randomizing the order in which the input unaligned sequences are concatenated. We also show how we select a single MSA from this set using a “majority voting” approach. We show that, on average, this data augmentation followed by majority voting approach provides a more accurate MSA than relying on a randomly sampled MSA from the set of alternative MSAs. The majority voting approach relies on computing for each MSA, the degree of its agreement with all other alternative MSAs and selecting the one that agrees the most (see Section 2.4).

##  

### 2.2 Generation of training and test data

We first describe in detail the simulation of nucleotide dataset SND1, in which each data point includes 10 unaligned sequences and their corresponding “true” MSA. We generated 395 000 and 3000 data points for training and testing data, respectively. For each data point, we sampled a random tree using the program ETE 3 (Huerta-Cepas *et al.* 2016), with tree lengths uniformly distributed in the range (0.05, 0.1). The sequences along each tree were simulated using SpartaABC (Loewenthal *et al.* 2021). Specifically, indel parameters were sampled from the following ranges: $R_{I},\;R_{D}\in (0.0,\;0.05)$, $A_{I},\;A_{D}\in \left(1.01,\;2.0\right)$, and root length ∈[32, 44]. Of note, the insertion ($R_{I}$ and $A_{I}$) and deletion ($R_{D}$ and $A_{D}$) model parameters were sampled independently allowing a rich-indel model, in which insertions and deletions can have different evolutionary dynamics. The above parameter ranges were found to accurately describe the indel evolution rates along the tree of life (Loewenthal *et al.* 2021). The WAG+G and the GTR+G substitution models were used for the protein and nucleotide datasets, respectively. The GTR+G frequencies were (0.37, 0.166, 0.307, 0.158) for the “T”, “C”, “A”, and “G”, respectively. Substitution rates were (0.444, 0.0843, 0.116, 0.107, 0.00027) for the “a”, “b”, “c”, “d”, and “e” rate parameters as defined in Yang (1994). These frequencies and rate parameters reflect those that characterize the Yeast Intron Database (Lopez and Séraphin 2000). Specific information for the simulation of each dataset is provided in Supplementary Table S2. The datasets are available on HuggingFace (Wolf *et al.* 2020) at: https://huggingface.co/dotan1111.

### 2.3 BetaAlign training and architecture

We applied the “vaswani_wmt_en_de_big” architecture (Vaswani *et al.* 2017) with 16 attention heads, embeddings size of 1024 and 6 layers. We also conducted an experiment to evaluate the effect of alternative architectures on performance (see Supplementary Information). We considered a variety of training hyperparameters configurations for the transformer, including different max tokens values, learning rates, and warmup updates and evaluated them on datasets of pairwise alignments (Supplementary Table S1). We continued to train two configurations that yielded the best results, which we denote as “original” and “alternative”. The max token parameter values were 4096 and 2048 for the original and alternative transformers, respectively. For both configurations, we used the same learning rate (5E-5) and warmup updates (3000). Model training and evaluations were executed on a Tesla V100-SXM2-32GB GPU machine.

### 2.4 Using alternative alignments to increase the accuracy of BetaAlign

**Figure 2.**
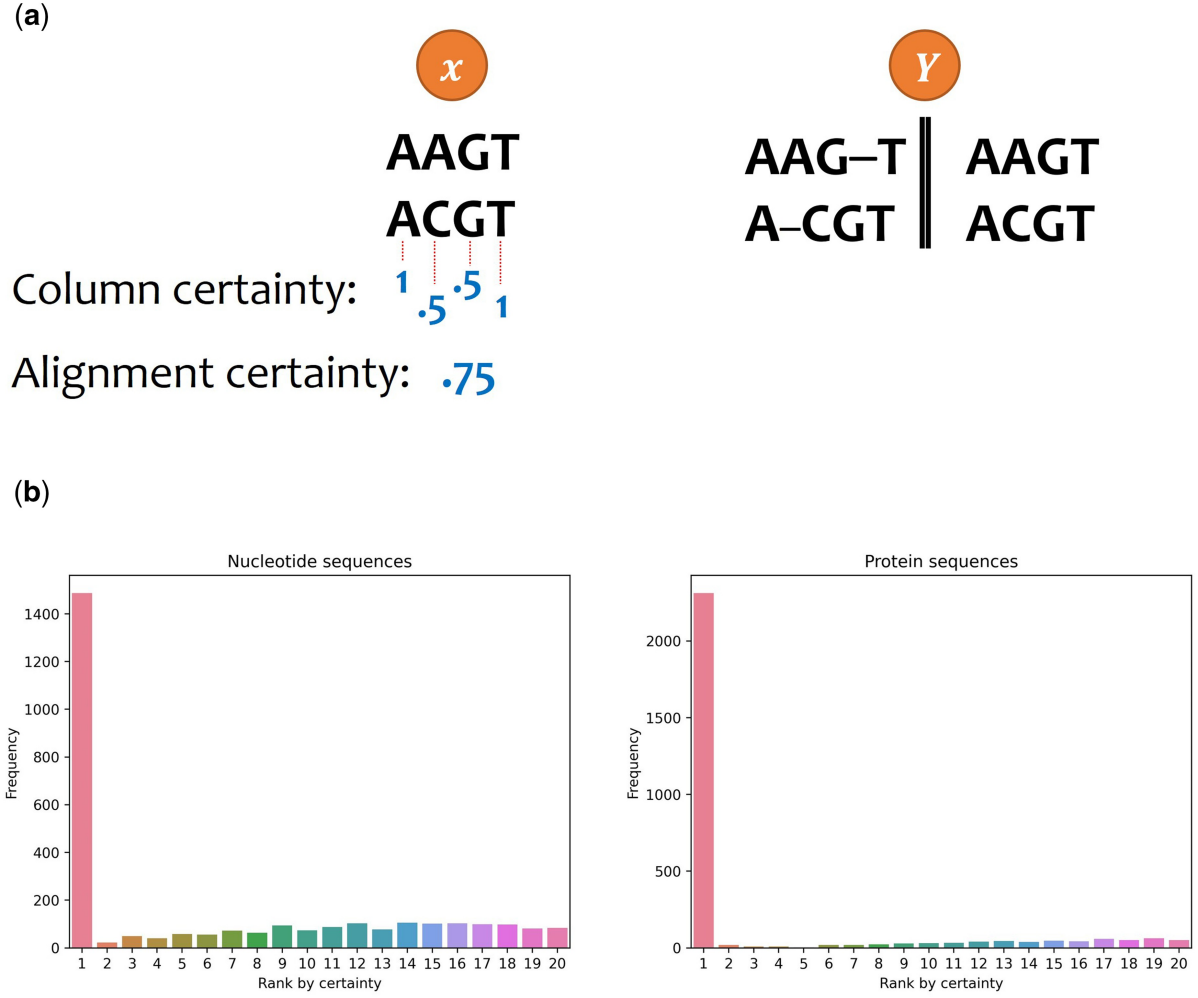
**Quantifying the correlation of alignment certainty and alignment accuracy.** (a) An illustration demonstrating the calculation of alignment certainty. Consider x to be a pairwise alignment where “AAGT” is aligned to “ACGT” and Y to be the collection of two alternative alignments: (i) where “AAG-T” is aligned to “A-CGT” and (ii) where “AAGT” is aligned to “ACGT”. To determine the certainty for each column in x, we count the number of its appearances in the set of alternative alignments Y and divide it by the size of the set Y. For example, the first column, “AA”, appears both in alignments (i) and (ii) and thus its certainty is 2/2. The second column in x, “AC” appears only in alignment (ii) and thus its certainty is 1/2. (b) The frequency of the optimal alternative alignment for each certainty rank. For each data point, a total of 20 alternative alignments were considered, each with 10 sequences (SND1 and SPD1 for the nucleotide and protein datasets, respectively). The 20 MSAs were ranked according to their certainty. Next, the most accurate MSA was detected (based on the CS accuracy score) and its rank recorded. Of note, some of the alternative MSAs may be identical. In case the most accurate MSA was ranked multiple times (e.g., the first and second ranks), we consider its rank to be the higher rank (e.g., the first). Shown is the distribution of ranks among 3000 independent data points. In almost all cases, the MSA that had the highest confidence is ranked highest.

A “column certainty” metric was employed to compute “alignment certainty”. Given an alignment, x, and a set of alternative alignments, Y, the column certainty of each column in x is the number of times the column appears in each alternative alignment y ∈ Y divided by the total number of alignments in Y. As a result, column certainty values range between 0 and 1, where a score of 1 indicates high certainty. The alignment certainty is defined as the average of the column certainty values (Fig. 2a).

It is possible to generate alternative MSAs for the same set of sequences. For example, alternative MSAs are generated by GUIDANCE to quantify the reliability of different regions within an MSA (Sela *et al.* 2015). These alternative MSAs are computed by considering alternative guide trees, considering co-optimal solutions of pairwise alignments, and changing the alignment scoring scheme. Alternative MSAs are also computed within the alignment program Muscle (Edgar 2022). The alignment that agrees best with the set of alternative MSAs is then chosen as the inferred MSA. We developed a similar approach for generating alternative MSAs, which is based on the deep learning methodology proposed here. Specifically, we alternate the order of the unaligned sequences given as input to the “*concat*” representation. This results in the inference of different MSAs for the same input. For example, an MSA of three sequences results in six different permutations, thus providing six alternative MSAs and similarly k! alternative alignments for k sequences. In addition, as we trained several transformers with different training parameters for each dataset, we can add alternative alignments from two or more transformers by processing the same input using these different transformers (Dotan *et al.* 2023).

Formally, let x and h be a list of unaligned sequences and an aligner program, respectively. When computing an alignment, the aligner is dependent on a set of parameters, i.e., a configuration, denoted by α. Altering α would output a different alignment for the same x and h. Thus, for a list of n different configurations: $\alpha_{1},\;\ldots ,\;\alpha_{i},\;\ldots ,\alpha_{n}$, one would receive n different alignments: ${Y=\{h}_{\alpha_{1}}\left(x\right),\;\ldots ,\;h_{\alpha_{i}}\left(x\right),\;\ldots ,\;h_{\alpha_{n}}\left(x\right)\}$. Of note, the alignments of different configurations could be the same. Creating the different configurations could be done by changing the scoring scheme for the aligners or by changing the permutation of the unaligned sequences in the case of BetaAlign (see above). For each alignment $h_{\alpha_{i}}\left(x\right),$ we calculate the alignment certainty described above, by comparing it to all the other alignments $Y\backslash \{h_{\alpha_{i}}\left(x\right)\}$ and computing the average number of shared columns. We return the alignment that maximizes the alignment certainty. Specifically, we have two transformer configurations (“original” and “alternative”) and for each, we generated 10 alternative MSAs. We return the valid alignment with the highest certainty.

### 2.5 Calculating the loss

The training loss is calculated using the cross-entropy loss function (Szegedy *et al.* 2016). Consider a specific position within a pairwise alignment. In the “*spaces*” representation, there are five possible tokens in the nucleotide output (the five characters, “A”, “C”, “G”, “T”, “–”. In fact, the pipe character can also appear, as we use the same dictionary as the input). Our aligner predicts (accounting for the proceeding predictions in the alignment) a probability for each token. Let $P_{i}\;$be the probability for the token in which the next character in the output alignment is *i*. Assume, for example that the correct class (the next character in the correct alignment) is “C”. In this case, the loss for this position is simply $-\log P_{C}$. The loss over the entire alignment string is the average loss over all positions in the alignment. If all positions are predicted correctly (i.e., with a $P_{i}\;\tilde{=}1$), the loss is close to zero. The higher the loss, the less accurate the prediction is. When the loss function is computed on the training data, we call it “training loss”, while when it is computed on the validation data, we call it “validation loss”.

### 2.6 Evaluating accuracy and coverage

We evaluated the performance of BetaAlign using two metrics: (i) column score (CS), which identifies how many columns are shared between the inferred and the true alignment. Of note, a shared column requires the same characters with the same positions of each character (Sela *et al.* 2015). The CS is the number of shared columns divided by the number of columns and thus the score is in the range [0,1]. The CS-error is the complementary of the CS to 1. (ii) We use the term coverage to denote the percentage of valid alignments out of the total number of MSAs generated by the transformer. Examples of invalid alignments are illustrated in Fig. 1c.

### 2.7 Evaluating the effect of training time and size

We generated datasets containing 50 000, 100 000, and 200 000 alignments. Next, we trained transformers on each of the datasets for 60 epochs with the original transformer training parameters. We evaluated the performance of the transformers at the end of each epoch, with respect to the following metrics: (i) training loss, (ii) validation loss, (iii) fraction of invalid alignments (i.e., 1—coverage), and (iv) CS-error. The validation data contained 2000 alignments, which were used to measure the validation loss. The test data contained 3000 alignments, which were used to measure the fraction of invalid alignments and CS-error. Of note, in each of the three experiments we initialized the model with random weights, and thus, transfer learning did not affect these results.

### 2.8 Evaluating the effect of indel parameters on alignment inference accuracy

To quantify the effect of the evolutionary parameters on alignment inference accuracy, we generated training and test data using the same random topology and branch lengths as were used in PD14 (see Supplementary Table S2). The range of indel evolutionary parameters was binned: For $A_{I}$ and $A_{D}$ that dictate indel-length distribution for insertions and deletions, respectively, the following ten bins were considered for each parameter: (1.0, 1.1), (1.1, 1.2) … (1.9, 2.0). For $R_{I}$ and $R_{D}$ that dictate indel rates relative to substitutions for insertions and deletions, respectively, the following ten bins were considered for each parameter: (0.000, 0.005), (0.005, 0.01) … (0.045, 0.05). We thus considered 100 bins for the pair ($A_{I},\;A_{D})$ and similarly for the pair ($R_{I}$, $R_{D}$). When analyzing the effect of $A_{I}$ and $A_{D}$, for each of the 100 ($A_{I},\;A_{D})$ bins, 100 alignments were generated, in which the $R_{I}$ and $R_{D}$ values were sampled randomly from the range (0.00, 0.05). Thus, in total 10 000 MSAs were considered when studying the effect of the $A_{I}$ and $A_{D}$ parameters. Similarly, 10 000 MSAs were considered when studying the effect of the $R_{I}$ and $R_{D}$ parameters, and in this case, in each MSA the $A_{I}$ and $A_{D}$ parameters were sampled from the range (1.0, 2.0). The score for each bin is the average over the scores of the 100 alignments in each bin.

### 2.9 Subspace learning evaluation

The MSA in the training data for BetaAlign is generated by evolving sequences along a specific phylogenetic tree and different MSAs are generated with different trees and with different evolutionary models. The substitution and indel dynamics are dictated in this simulation by an evolutionary model (a continuous-time Markov process). Let g be the set of evolutionary models and trees used to generate the data. Clearly, a trained aligner, *h*, depends on g. In other words, our aligner learns to align sequences generated by the set of evolutionary models g that generated the training data. Thus, we can easily create aligners that will best suit a specific subspace of model parameters and trees, e.g., aligners for a specific phylogenetic tree, and similarly aligners for species or proteins with a specific indel or substitution dynamics. In subspace learning, the transformer is optimized on a subspace of the alignment parameters space. To test how subspace learning affects performance, we generated three nucleotide datasets, each one with a narrower range of model parameters, i.e., $A_{I}$, $A_{D}$, $R_{D}$, and $R_{I}$, branch lengths and root lengths (ND10, ND11, and ND12). We trained BetaAlign starting with the dataset of the widest parameter range (ND10), which we named “general”. Then, the optimized transformers were used as the starting point for additional training on the next dataset, ND11, whose model parameters are a subset of those of ND10. We named this dataset “specific”. The optimized transformers from ND11 were then further trained on the next dataset (ND12) “ultra specific”. Each of the three transformers was evaluated on each of the three test datasets.

### 2.10 Embedding of MSAs in a high-dimensional space

The deep learning approach presented here enables embedding the information within the sequences in a high-dimensional space, i.e., it allows automatic feature extraction, which could be utilized for downstream analyses. The high-dimensional vector is created within the encoding process from a set of unaligned sequences. To obtain the embedded vector, the unaligned sequences were given as an input to the trained transformer. The vector is internally created by the encoder part of the transformer, and we have modified the code of the transformer to extract it (to reduce running time, we skipped the decoder step). This high-dimensional vector contains ∼1024 ×n×l entries, where *n* is the number of input sequences and *l* is the average length of unaligned sequences. A representation of this vector, for three sequences, is given in Supplementary Fig. S2a.

For various downstream tasks, it is often desirable to compress this vector to a fixed size, i.e., a size that does not depend on the sequence length (the compressed vector size does depend on the number of sequences). For the compression example shown in Supplementary Fig. S2, the uncompressed vector is of size 1024×15 and the size of the compressed vector is 1024×5. Each of the unaligned sequences is represented by 1024 entries in the compressed vector by row-wise averaging of the corresponding tokens in the input sequences. In addition, we use the representations of the pipe character in the compressed vector. Thus, the compressed vector corresponds to a vector of a fixed size of 1024 × (2n – 1).

### 2.11 Evaluating and implementing transfer learning

In our work, transfer learning was repeatedly used for training the transformers. The first protein transformer was trained on a simple dataset of pairwise amino acid sequences (we denote this dataset PD1, for protein dataset 1). Its weights were randomly sampled with default values of the Fairseq library (Ott *et al.* 2019). The resulting trained transformer is termed as “PT1”, for protein transformer 1. PT1 was next trained on PD2, resulting in PT2, etc. The term transfer learning is used to denote the fact that in order to obtain PT2, the transformer trained on PD2 was initialized with weights transferred from PT1, rather than random initialization. A similar process was used to train the nucleotide-based transformers (NT1, NT2, etc.) on nucleotide datasets (ND1, ND2, etc.). Of note, transfer learning was applied across this study only between models that processed data with the same representation, i.e., they share the same dictionaries.

**Figure 3.**
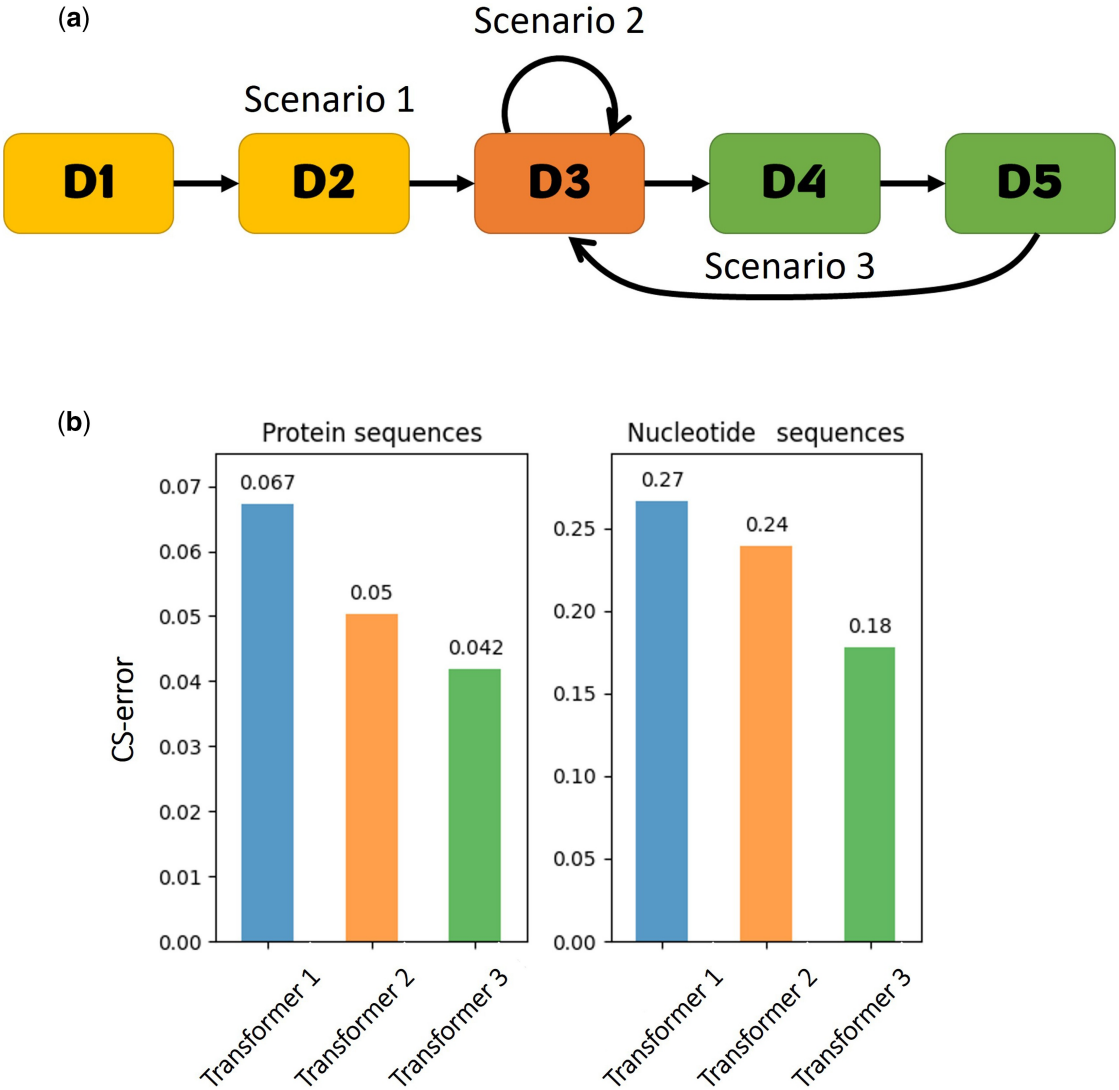
**Quantifying the contribution of transfer learning to performance.** (a) The transfer learning path. Scenario 1 includes training on “D1”, “D2”, and “D3”. Scenario 2 is the same as Scenario 1, but the transformer was trained twice on “D3”. Scenario 3 includes training on “D1”, “D2”, “D3”, “D4”, “D5” and then again on “D3”. “D1” and “D2” represent simpler datasets. “D3” is the target dataset, composed of MSAs of three DNA or amino acid sequences, on which the performance was evaluated. “D4” and “D5” represent more complex datasets. Arrows between datasets represent the transfer learning path, i.e., the transformer optimized on a dataset was used as a base transformer for the next dataset. (b) The effect of transfer learning on the performance.

We aimed to evaluate the contribution of transfer learning. To this end, we compared three different scenarios (illustrated in Fig. 3). In Scenario 1, we evaluate a transformer that first encounters protein data PD5 (three protein sequences). This transformer was trained before on simpler datasets. In Scenario 2, the trained transformer from Scenario 1 was retrained on PD5, without experiencing more complex datasets. In Scenario 3, the trained transformer from Scenario 1 was trained on additional more complex datasets (PD6, PD7, PD8, PD9, PD10, PD11, PD12, PD13, PD14, and PD15) and was then retrained on PD5.

A similar evaluation was done on nucleotide transformers. Here instead of PD5, the base-dataset was ND4, comprised of alignments of three sequences. In Scenario 3, the additional more complex datasets are: ND5, ND6, ND7, ND8, ND9, ND10, ND11, ND12, ND13, and ND14.

### 2.12 Comparing against other alignment programs

The performance of BetaAlign was compared to the following programs used with default parameters: MUSCLE v3.8.1551 (Edgar 2004), MAFFT v7.475 (Katoh and Standley 2013), PRANK v.150803 (Löytynoja and Goldman 2008), ClustalW 2.1 (Larkin *et al.* 2007), and DIALIGN dialign2-2 (Morgenstern 2004). Specific commands used for evaluation are provided in the Supplementary Information.

## 3 Results

### 3.1 Effect of training time and size

**Figure 4.**
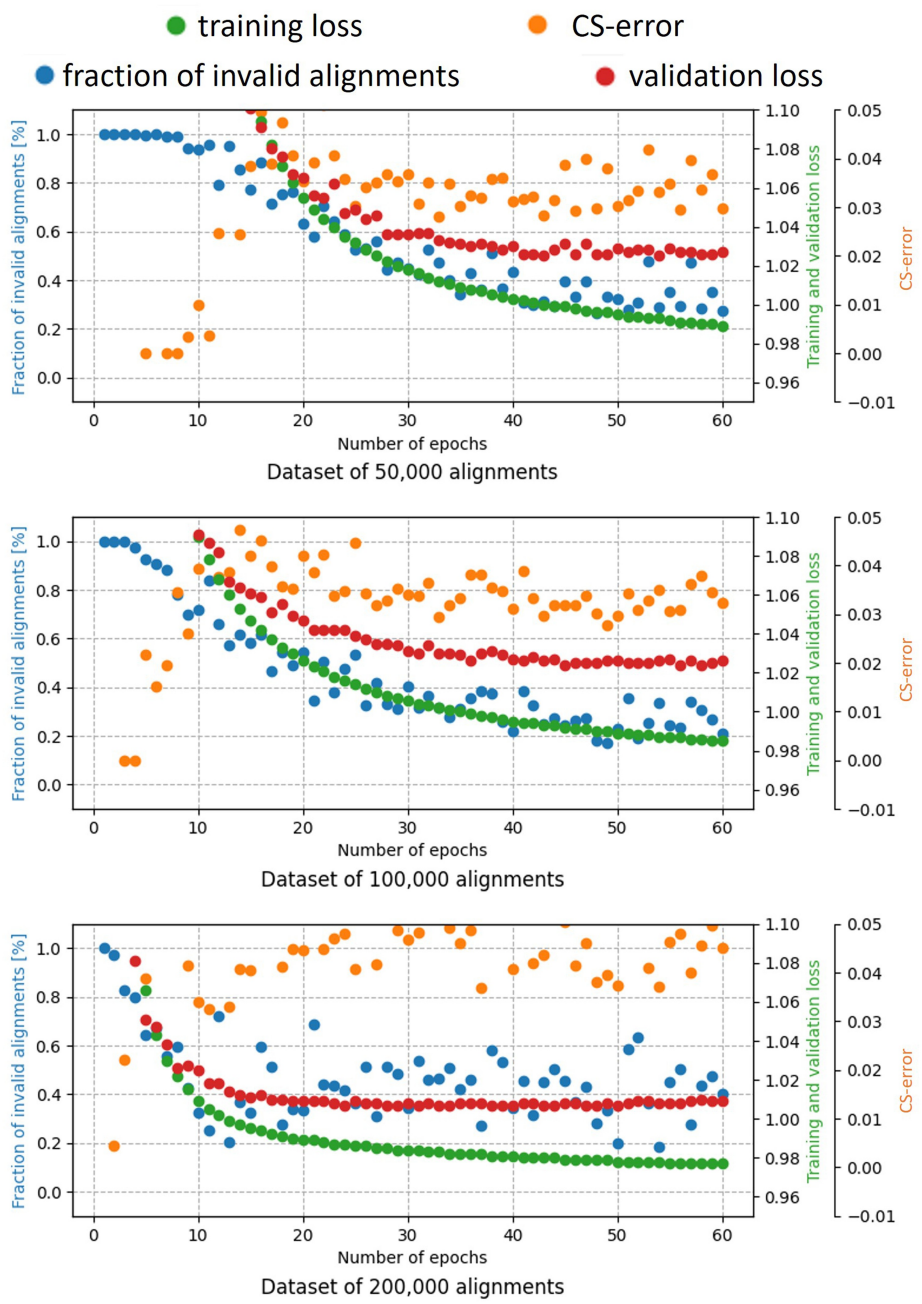
**Effect of increasing the training time (number of epochs) and size (number of different MSAs) on the fraction of invalid alignments (blue dots), CS-error (orange dots), validation loss (red dots), and training loss (green dots).** All alignments were of three protein sequences, dataset SPD2. Note that the figure contains the four metrics together for comparing the correlation between the metrics. Each metric has a different range, and thus, there are multiple *y*-axes. Also note that the errors and coverage in this graph are based on a single alternative alignment, while in practice both the accuracy and coverage are substantially improved by considering a set of alternative MSAs (see text for details). The training loss and the validation loss clearly decrease with the number of epochs and the number of alignments used for training. In contrast, the CS-error and the fraction of invalid alignments show mediocre correlations with the training loss, and do not significantly decrease with the number of alignments used for training.

We tested how the number of epochs (a single pass on the whole training set) and training size affect the accuracy and coverage of BetaAlign. We compared the model’s performance when trained on three training data sizes: 50 000, 100 000, and 200 000 protein alignments. Our results clearly indicate that for all datasets, the training loss (see Section 2.5) decreases as the number of epochs increases, reaching almost a plateau when the data size is 200 000 alignments (Fig. 4). For each training data size, the validation loss follows the decrease in the training loss, suggesting that there is no overfitting for the transformer. The coverage (fraction of resulting alignments that are valid) also continuously increases, e.g., after 20 epochs the coverage was ∼40%, while after 60 epochs, the coverage was already ∼80%.

Inference accuracy is measured using the column score (CS), which quantifies the number of columns that are shared between the inferred and the “true” MSA (see Section 2.6). The CS-error (one minus the CS) seems to substantially fluctuate even after 30 epochs (we note that the CS-error quantifies the error on valid alignments only, while the loss function quantifies the error on all alignments). The correlation between the loss on the validation data and the CS-error on the dataset of 100 000 alignments, between epochs 20 and 60, was $R^{2}$ = 0.467 (*p* = 0.0023). This correlation suggests that reducing the loss also reduces the CS-error, despite the clear differences between these two functions.

Comparing the training and validation loss between the different training size datasets indicated that increasing the training size decreases the loss as expected (training loss at epoch 60: 0.989, 0.985, 0.977, for datasets of 50 000, 100 000, 200 000, respectively). This gain in accuracy as reflected in the loss function was not evident when the performance is measured by the CS-error, reflecting lack of strong correlation between these two scores.

### 3.2 Effect of indel model parameters on BetaAlign performance

We next studied the effect of the different indel parameters (of the assumed indel model that generated the simulated data) on the performance. To this end, we divided the alignments into bins by their evolutionary parameters: the insertion and deletion rate parameters ($R_{I}$ and $R_{D}$, respectively) and the parameters that determine the distribution of indel lengths ($A_{I}$ and $A_{D}$ for the insertion and deletion distributions, respectively). As expected, increasing the indel rate parameters $R_{I}$ and $R_{D}$ substantially decreases accuracy (Supplementary Fig. S1a). The size distribution of the indels had little effect on accuracy (Supplementary Fig. S1b).

### 3.3 Subspace learning

**Figure 5.**
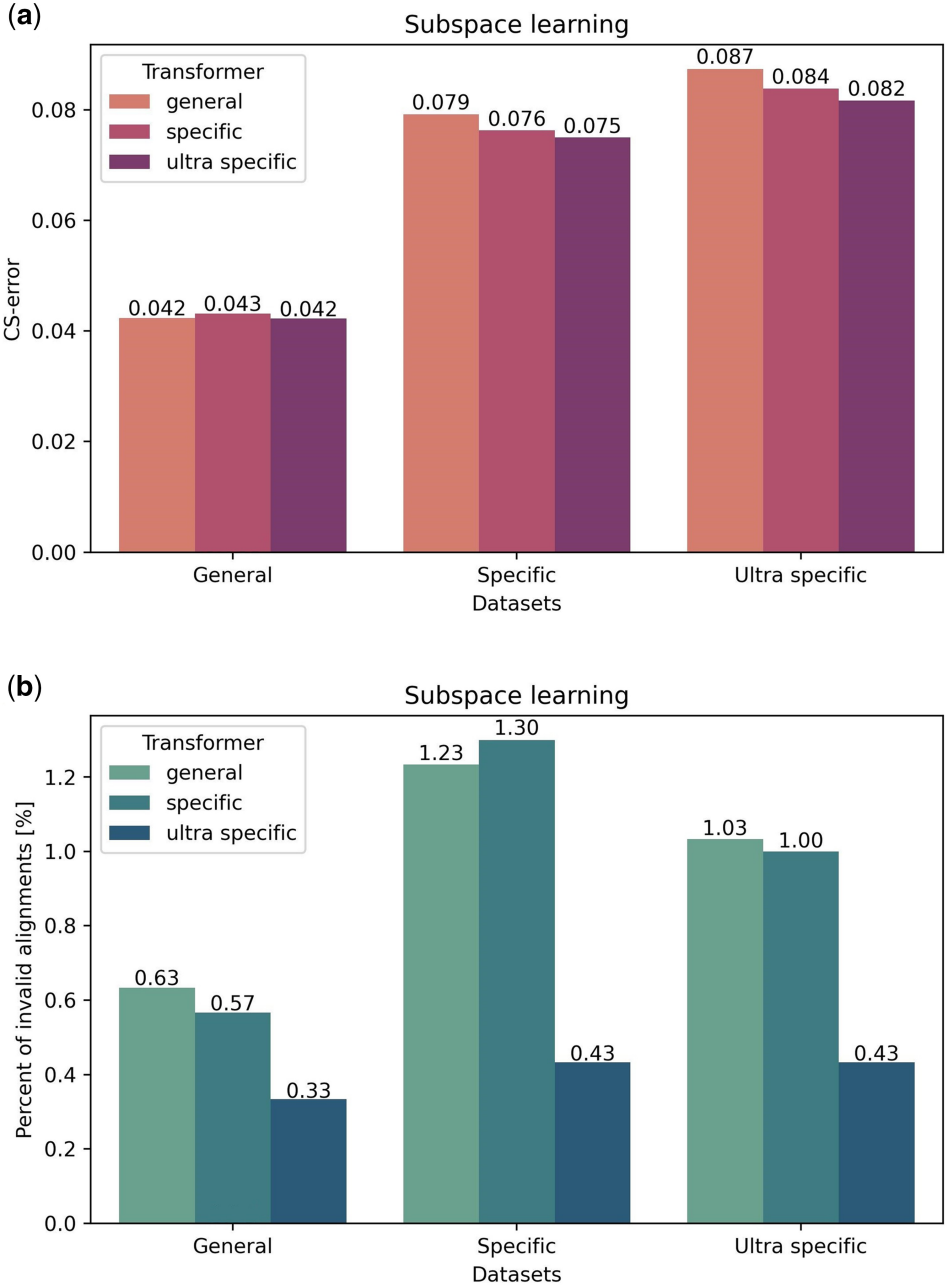
**Effect of subspace learning on the CS-error (a) and the fraction of invalid alignments (b).** The three transformers: “general”, “specific”, and “ultra specific” were trained on the “general”, “specific”, and “ultra specific”, datasets, respectively. The “ultra specific” dataset (ND12) parameters (e.g., the indel rates) are a subset of the “specific” dataset (ND11) parameters, which are a subset of the “general” dataset (ND10) parameters. The difference between the accuracy of “general” and “ultra specific” transformers on the “ultra specific” dataset is significant (paired *t*-test; p<0.05).

As stated above, we can train a transformer on a set of MSAs that share specific features, e.g., training them on MSAs with a high deletion rate and a low insertion rate. Deep learning models have a large number of free parameters, allowing learning complex patterns. In subspace learning, we optimize these free parameters again on a subset of the dataset. The starting weights of the parameters are the weights obtained for the entire range. Nevertheless, we note that as the architecture is fixed, the number of free parameters is fixed as well. To determine if such a subspace-learning approach increases accuracy, we simulated three nucleotide datasets of five sequences per sample (see Section 2.9). The first dataset, “general” (ND10), was simulated with a wide range of indel model parameters. The second dataset, “specific” (ND11), was simulated on a subspace of the indel model parameter space, i.e., the generated MSAs resemble each other in terms of indel dynamics. Finally, the third dataset, “ultra specific” (ND12), is even more restrictive in terms of the allowed indel dynamics (see Supplementary Table S2). Our results suggest that subspace learning can improve both coverage and accuracy (Fig. 5), with a more substantial effect on coverage. This highlights the importance of fitting the correct configuration of the alignment program (and in our case the training of the transformer) to the specific data. These results demonstrate that subspace learning has the potential to improve the accuracy of BetaAlign.

### 3.4 Embedding extraction for downstream tasks

**Figure 6.**
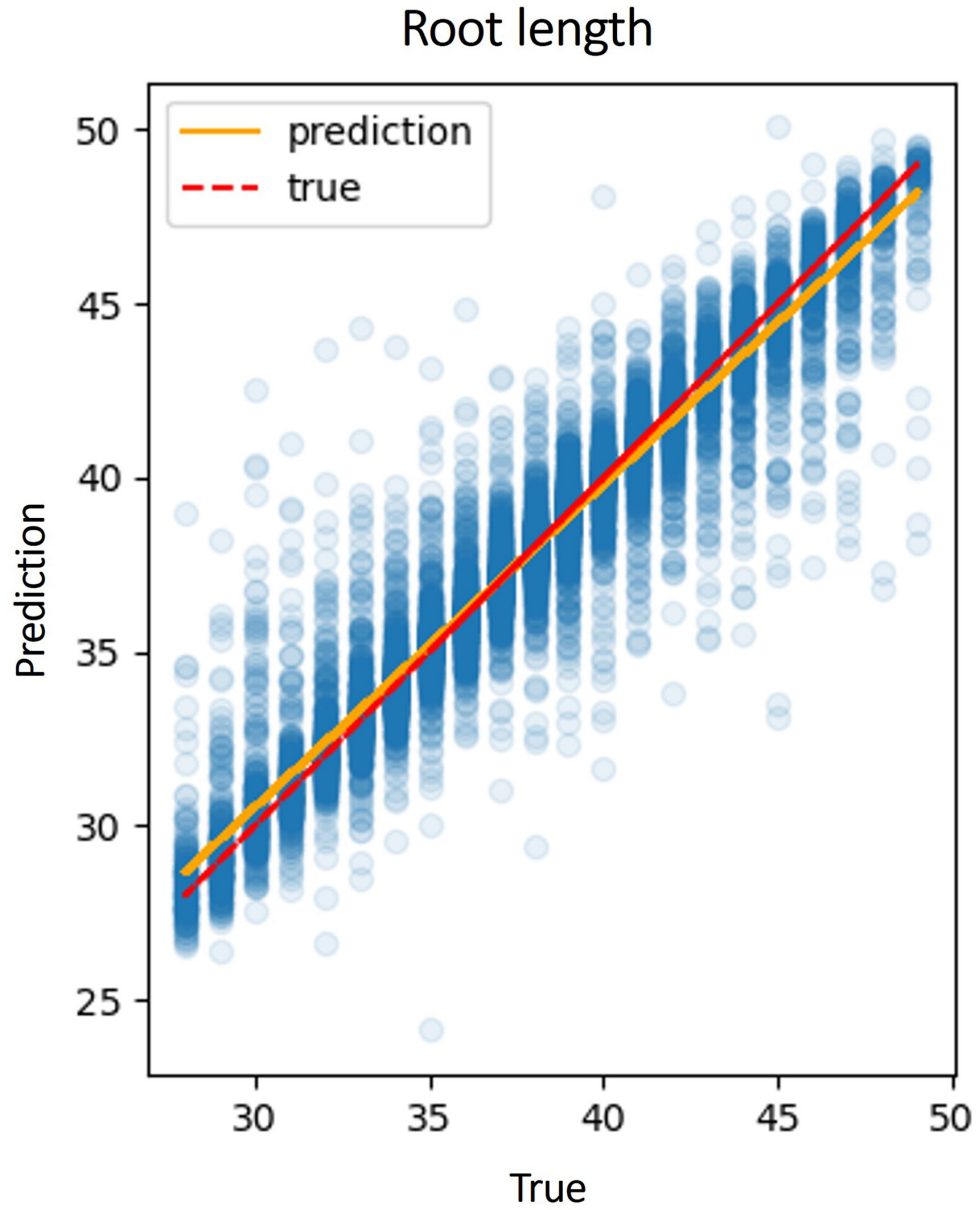
**Results of the linear regressor trained to predict the root length from the embedding of the unaligned sequences, with an**  $R^{2}$  **of**  0.91  **and**  MSE  **of 2.003 base pairs.** The solid (orange) line is the regression line and the dashed (red) line reflects the Y=x function. The embeddings are of the ND10 dataset sequences.

Transformers are composed of two parts, the encoder and the decoder. The encoder creates high-dimensional vector representations of the source sentence, i.e., the unaligned sequences, which are passed to the decoder to create the translated sentence, i.e., the aligned sequences. This high-dimensional vector embeds the information in sequences as a numeric representation. We compressed this vector to a vector of a size that does not depend on the number of positions. In the case of *n* sequences, the dimension of the vector is $1024\times \left(2n-1\right)$ (see Section 2.10). To exemplify the utility of such a representation, we used this vector representation as input for a different machine-learning task, which is to estimate for each MSA the length of the root sequence, from which the resulting sequences diverged. To this end, we trained a linear regression model that takes the coordinates of the compressed high-dimensional vector as input. The training set includes 90 000 nucleotide MSAs, each with five sequences (ND10). The accuracy of the linear-regression model using these features was evaluated on test data comprising 10 000 MSAs (Fig. 6). The significant correlation between the true and inferred root lengths [$R^{2}=0.91$ and 2.003 base pairs mean squared error (MSE)] suggests that our approach can be used to compactly code sequences, as a preliminary step for downstream machine-learning tasks.

### 3.5 Transfer learning

Our approach heavily depends on transfer learning. Except for the first transformers, for which the weights were randomly initialized, all other transformers used initial weights that were optimized on a previous dataset. The transformers of the nucleotide datasets have a different path of training from the transformers of the amino acid datasets. In addition, each transformer is optimized based on the previous transformer with the same configuration (as we trained two different transformers for each dataset). To evaluate the contribution of transfer learning to performance, we tested three alternative scenarios (Fig. 3a, see Section 2.11). Briefly, the transformer in Scenario 1 (Transformer 1) is trained once on a target dataset. Transformer 2 (Scenario 2) started from the end point of Transformer 1 and was retrained on the same target dataset. Transformer 3 (Scenario 3) started from the end point of Transformer 1 and was trained on various other datasets, and then retrained on the same target dataset. Our results demonstrated the benefit of transfer learning (Fig. 3b). Transformer 3 outperformed Transformer 1, both for protein and DNA sequences, with error reduction of 37.3% and 33.3%, respectively (paired *t*-test; *p* < 0.005). It may be that the increased accuracy resulted from the fact that Transformer 3 was trained twice on the target dataset and not due to the additional training. To test this hypothesis, we compared it to Transformer 2. Our analysis suggests that some of the improved accuracy is indeed due to the extra training (comparing Transformers 1 and 2). Nevertheless, it also shows that transfer learning substantially contributes to performance (comparing Transformers 2 and 3), resulting in 16% and 25% error reductions for protein and DNA, respectively (paired *t*-test; *p* < 0.005).

### 3.6 Correlation of certainty and the alignment accuracy

We found a strong dependence between the alignment certainty and the CS-score (Fig. 2). As the certainty of alignments can be calculated by creating multiple alternative alignments for the same set of unaligned sequences (see Section 2.4), we could utilize this dependence to infer the most accurate alignment, similar to a previous approach (Edgar 2022).

Having observed that the alignment with the highest (alignment) certainty is ranked higher than expected (among the set of alternative alignments from a specific dataset), we next directly compared performance between choosing the alignment alternative with the highest certainty and selecting the first alternative alignment. We tested this approach on 10-sequences data points (SND1 and SPD1) and observed a significant CS-error reduction of 9.8% and 20.9% for DNA and protein alignments, respectively (paired *t*-test; p = 0.002).

### 3.7 Comparing performance

**Figure 7.**
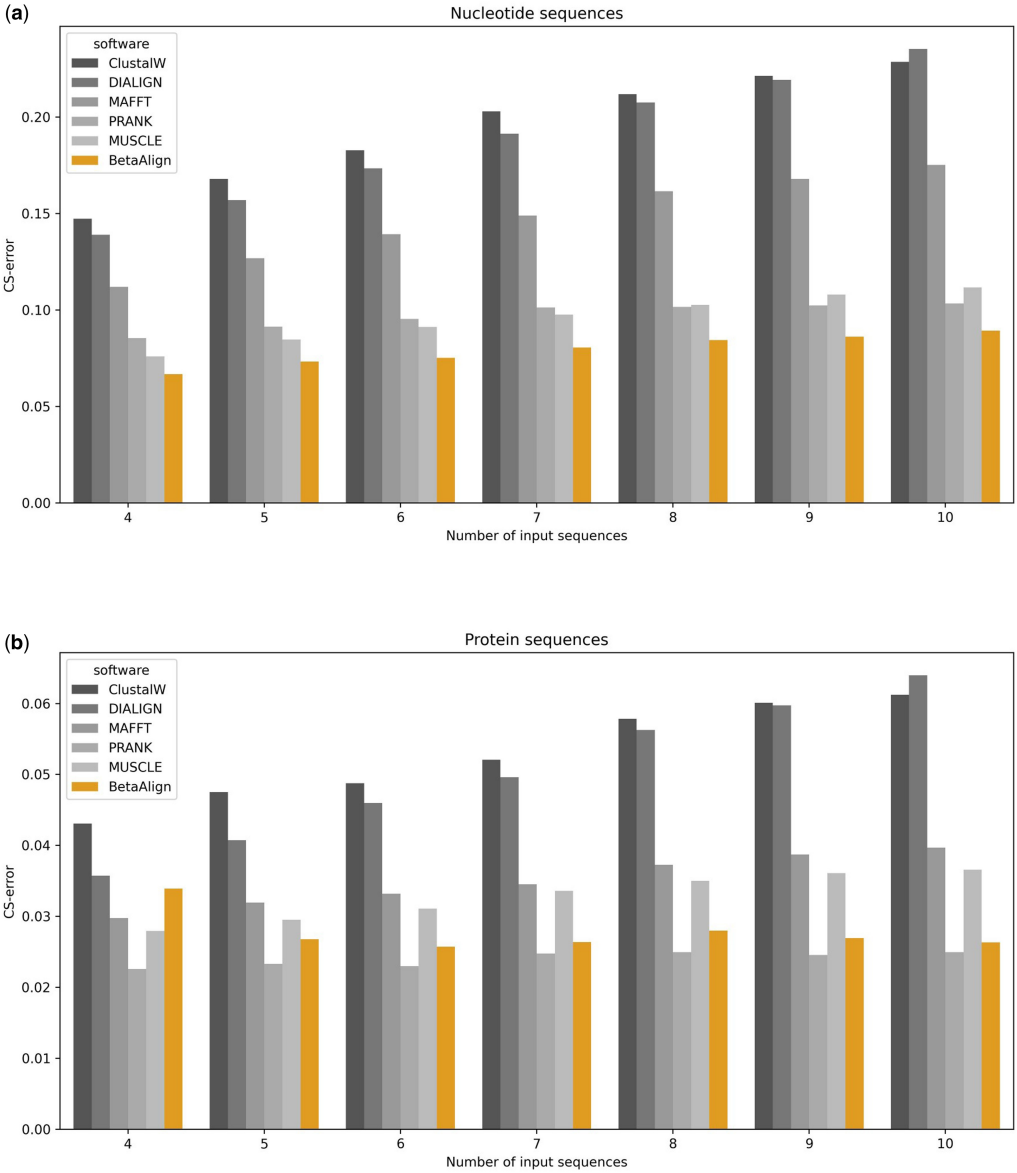
**Comparing the results of BetaAlign with different aligners on SND1 (a) and SPD1 (b).** The *y*-axis represents the performance of sequence alignment programs. The lower the CS-error, the better the performance.

We compared the performance of BetaAlign after selecting the MSA with the highest certainty against other commonly used alignment programs, both for DNA and protein sequences (Fig. 7). For DNA sequences, regardless of the number of sequences analyzed, BetaAlign was the most accurate (paired *t*-test; $p<10^{-7}$), with a minimal error reduction of 12.7%. The second most accurate alignment program was MUSCLE for 4–7 sequences and PRANK for 8–10 sequences. For 10 sequences, for example, BetaAlign had an 13.7% error reduction compared to PRANK (paired *t*-test; $p<10^{-12}$) and similar results were obtained for other number of sequences. MAFFT, DIALIGN, and ClustalW had a significantly lower performance, with MAFFT outperforming the two other alignment programs. Notably, for protein sequences, BetaAlign was typically the second most accurate. For 10 sequences, the error reduction of PRANK was 5.1% relative to BetaAlign. We speculate that the higher accuracy of protein MSAs compared to DNA-based MSAs, which is observed across all methods, stems from the higher alphabet size of protein sequences, which makes it easier to find anchors to guide MSA inference.

## 4 Discussion

The weights that are learned by the encoder can be used as a starting point for other machine-learning tasks, i.e., the sequences are embedded as meaningful vectors that hold contextual information. In this work, we demonstrated using such embedding for predicting the length of ancestral sequences, without computing the MSA. A similar approach can be used for other machine-learning tasks, e.g., secondary structure prediction, predicting the stability of proteins, and ancestral sequence reconstruction. In NLP, transferring representations from one task to another is highly common, and encoder–decoder models are commonly used for this purpose (McCann *et al.* 2017).

There are limitations when using NLP approaches for sequence alignment, one of which arises from the maximum sequence length that can be inserted into an attention-based model. This limitation stems from computing attention matrices, in which the memory requirement increases quadratically with the total length of the input and output sequences. The data size that can be processed depends on multiple factors that dictate the size of the obtained alignment. These factors include the number of sequences, the root length, the sequence divergence, which is dictated by the edges of the phylogenetic tree, and the indel rates and indel-length distributions. In general, the memory limitation is computer-specific, and on the current GPUs that we have used, we could analyze 1024 tokens (e.g., ∼10 sequences of 100 aligned columns, see Section 2). To overcome this issue, we have developed a novel approach that involves splitting and merging the alignment while training the transformer on a slightly different task (Dotan *et al.* 2023). It is possible to apply different techniques to increase the limit on the sizes of the sequences. For example, a different tokenization technique allows multiple amino acids or nucleotides to be considered as a single token, and thus reduces the number of tokens for the entire sequence (Dotan *et al.* 2024). Another option would be to employ state-space models instead of transformers (Gu *et al.* 2022).

Our proposed method introduces a paradigm shift: it redirects the focus from the traditionally labor-intensive task of developing new sequence aligners to the more manageable process of creating simulations that replicate the evolutionary dynamics observed in empirical data. This approach is particularly beneficial for incorporating additional types of evolutionary events. For example, developing an aligner capable of detecting inversions in unaligned sequences would be complex and likely increase the algorithm’s complexity. In contrast, BetaAlign can be easily trained on simulated data that include inversions, enabling it to effectively align sequences which experience inversions.

Generating multiple alternative alignments can be important for various applications, including the inference of alignment reliability (Sela *et al.* 2015). MergeAlign (Collingridge and Kelly 2012) combines alternative alignments into a single consensus, offering a promising method for enhancing the accuracy and reliability of MSAs. Multiple alternative MSAs are also accounted for in Bayesian alignment strategies, such as Bali-Phy (Redelings 2021). However, Bayesian methods rely on a predefined prior and stochastic evolutionary model to guide alignment calculations, while in BetaAlign, the stochastic method is used for generating the training data, and not for the alignment inference.

We have coupled the NLP domain and the MSA problem by using transformers that were originally designed for natural languages. Future improvements in the NLP field are likely to directly impact future alignment methodologies. We expect that transformers that are dedicated to the task of sequence alignment, together with other breakthroughs in machine learning, will lead to alignment algorithms that account for the specific grammar rules of each set of analyzed sequences and will substantially outperform existing aligners.

## Supplementary Material

btaf009_Supplementary_Data

## References

[btaf009-B1] Ajawatanawong P , Baldauf SL. Evolution of protein indels in plants, animals and fungi. BMC Evol Biol 2013;13:140. 23826714 10.1186/1471-2148-13-140 PMC3706215

[btaf009-B2] Avram O , Durmus B, Rakocz N et al SLIViT: a general AI framework for accurate clinical-feature diagnosis from limited 3D medical-imaging data. Invest Ophthalmol Vis Sci 2024;65:1614.

[btaf009-B3] Bahdanau D , Cho K, Bengio Y. Neural machine translation by jointly learning to align and translate. In: International Conference on Learning Representations (ICLR), San Diego, CA, USA, 2015.

[btaf009-B4] Chang J-M , Di Tommaso P, Notredame C. TCS: a new multiple sequence alignment reliability measure to estimate alignment accuracy and improve phylogenetic tree reconstruction. Mol Biol Evol 2014;31:1625–37. 24694831 10.1093/molbev/msu117

[btaf009-B5] Collingridge PW , Kelly S. MergeAlign: improving multiple sequence alignment performance by dynamic reconstruction of consensus multiple sequence alignments. BMC Bioinformatics 2012;13:117. 22646090 10.1186/1471-2105-13-117 PMC3413523

[btaf009-B6] Dotan E , Belinkov Y, Avram O et al Multiple sequence alignment as a sequence-to-sequence learning problem. In: International Conference on Learning Representations (ICLR), Kigali, Rwanda, 2023.

[btaf009-B7] Dotan E , Jaschek G, Pupko T et al Effect of tokenization on transformers for biological sequences. Bioinformatics 2024;40:4:btae196. 10.1093/bioinformatics/btae196 PMC11055402 38608190

[btaf009-B8] Edgar RC. MUSCLE: multiple sequence alignment with high accuracy and high throughput. Nucleic Acids Res 2004;32:1792–7. 15034147 10.1093/nar/gkh340 PMC390337

[btaf009-B9] Edgar RC. Muscle5: high-accuracy alignment ensembles enable unbiased assessments of sequence homology and phylogeny. Nat Commun 2022;13:6968. 36379955 10.1038/s41467-022-34630-w PMC9664440

[btaf009-B10] Gu A , Goel K, Ré C. Efficiently modeling long sequences with structured state. In: *International Conference on Learning Representations (ICLR)*, Virtual Event, 2022.

[btaf009-B11] Huerta-Cepas J , Serra F, Bork P. ETE 3: reconstruction, analysis, and visualization of phylogenomic data. Mol Biol Evol 2016;33:1635–8. 26921390 10.1093/molbev/msw046 PMC4868116

[btaf009-B12] Iantorno S , Gori K, Goldman N et al Who watches the watchmen? An appraisal of benchmarks for multiple sequence alignment. Methods Mol Biol. 2014;1079:59–73. 24170395 10.1007/978-1-62703-646-7_4

[btaf009-B13] Katoh K , Standley DM. MAFFT multiple sequence alignment software version 7: improvements in performance and usability. Mol Biol Evol 2013;30:772–80. 23329690 10.1093/molbev/mst010 PMC3603318

[btaf009-B14] Larkin MA , Blackshields G, Brown NP et al Clustal W and clustal X version 2.0. Bioinformatics 2007;23:2947–8. 17846036 10.1093/bioinformatics/btm404

[btaf009-B16] Loewenthal G , Rapoport D, Avram O et al A probabilistic model for indel evolution: differentiating insertions from deletions. Mol Biol Evol 2021;38:5769–81. 34469521 10.1093/molbev/msab266 PMC8662616

[btaf009-B17] Lopez PJ , Séraphin B. YIDB: the yeast intron DataBase. Nucleic Acids Res 2000;28:85–6. 10592188 10.1093/nar/28.1.85 PMC102386

[btaf009-B18] Löytynoja A. Phylogeny-aware alignment with PRANK. Methods Mol Biol 2014;1079:155–70. 24170401 10.1007/978-1-62703-646-7_10

[btaf009-B19] Löytynoja A , Goldman N. Phylogeny-aware gap placement prevents errors in sequence alignment and evolutionary analysis. Science 2008;320:1632–5. 18566285 10.1126/science.1158395

[btaf009-B20] McCann B , Bradbury J, Xiong C et al Learned in translation: contextualized word vectors. Adv Neural Inf Process Syst 2017;30:6297–308.

[btaf009-B21] Morgenstern B. DIALIGN: multiple DNA and protein sequence alignment at BiBiServ. Nucleic Acids Res 2004;32:W33–6. 15215344 10.1093/nar/gkh373 PMC441511

[btaf009-B22] Needleman SB , Wunsch CD. A general method applicable to the search for similarities in the amino acid sequence of two proteins. J Mol Biol 1970;48:443–53. 5420325 10.1016/0022-2836(70)90057-4

[btaf009-B23] Ott M , Edunov S, Baevski A et al fairseq: a fast, extensible toolkit for sequence modeling. In: *Proceedings of the 2019 Conference of the North American Chapter of the Association for Computational Linguistics (Demonstrations), Minneapolis, Minnesota*. p.48–53. Association for Computational Linguistics, 2019. https://aclanthology.org/N19-1000/

[btaf009-B24] Pesole G , Gissi C, De Chirico A et al Nucleotide substitution rate of mammalian mitochondrial genomes. J Mol Evol 1999;48:427–34. 10079281 10.1007/pl00006487

[btaf009-B25] Redelings BD. BAli-Phy version 3: model-based co-estimation of alignment and phylogeny. Bioinformatics 2021;37:3032–4. 33677478 10.1093/bioinformatics/btab129

[btaf009-B26] Sela I , Ashkenazy H, Katoh K et al GUIDANCE2: accurate detection of unreliable alignment regions accounting for the uncertainty of multiple parameters. Nucleic Acids Res 2015;43:W7–14. 25883146 10.1093/nar/gkv318 PMC4489236

[btaf009-B27] Shalumov V , Haskey H. HeRo: RoBERTa and Longformer Hebrew language models. arXiv, https://arxiv.org/abs/2304.11077, 2023, preprint: not peer reviewed.

[btaf009-B28] Sutskever I , Vinyals O, Le QV. Sequence to sequence learning with neural networks. Adv Neural Inf Process Syst 2014;27:3104–12.

[btaf009-B29] Szegedy C , Vanhoucke V, Ioffe S et al Rethinking the Inception Architecture for Computer Vision. In: *Proceedings of the IEEE Conference on Computer Vision and Pattern Recognition, pp 2818–2826*. 2016.

[btaf009-B30] Tan C , Sun F, Kong T et al A survey on deep transfer learning. In: *Artificial Neural Networks and Machine Learning—ICANN 2018, Rhodes, Greece,* 2018.

[btaf009-B31] Vaswani A , Shazeer N, Parmar N et al Attention is all you need. In: *31st Conference on Neural Information Processing Systems (NIPS), Long Beach, CA, USA*, 2017.

[btaf009-B32] Walker CR , Scally A, Maio ND et al Short-range template switching in great ape genomes explored using pair hidden markov models. PLoS Genet 2021;17:e1009221. 33651813 10.1371/journal.pgen.1009221 PMC7954356

[btaf009-B33] Wang H-C , Li K, Susko E et al A class frequency mixture model that adjusts for site-specific amino acid frequencies and improves inference of protein phylogeny. BMC Evol Biol 2008;8:331. 19087270 10.1186/1471-2148-8-331 PMC2628903

[btaf009-B34] Wang L , Jiang T. On the complexity of multiple sequence alignment. J Comput Biol 1994;1:337–48. 8790475 10.1089/cmb.1994.1.337

[btaf009-B35] Wolf T , Debut L, Sanh V et al Transformers: state-of-the-art natural language processing. In: *Proceedings of the 2020 Conference on Empirical Methods in Natural Language Processing: System Demonstrations*. p.38–45, Virtual Event, 2020.

[btaf009-B36] Wolf Y , Madej T, Babenko V et al Long-term trends in evolution of indels in protein sequences. BMC Evol Biol 2007;7:19. 17298668 10.1186/1471-2148-7-19 PMC1805498

[btaf009-B37] Yang Z. Estimating the pattern of nucleotide substitution. J Mol Evol 1994;39:105–11. 8064867 10.1007/BF00178256

