# Supplementary material for: BetaAlign: a deep learning approach for multiple sequence alignment
Source: Bioinformatics. 2025 Jan 8;41(1):btaf009. doi: 10.1093/bioinformatics/btaf009 (PMC11758787; doi:10.1093/bioinformatics/btaf009)
Supplement: btaf009_Supplementary_Data [file btaf009_supplementary_data.zip › 2873e_??SupplementaryInformation_v6.docx]

# Supplementary Information

**BetaAlign: a deep learning approach for multiple sequence alignment**

Edo Dotan^1,2^, Elya Wygoda^1^, Noa Ecker^1^, Michael Alburquerque^1^, Oren Avram^3^, Yonatan Belinkov^2,^†, and Tal Pupko^1,^†

^1^ The Shmunis School of Biomedicine and Cancer Research, George S. Wise Faculty of Life Sciences, Tel Aviv University, Tel Aviv 69978, Israel.

^2^ The Henry and Marilyn Taub Faculty of Computer Science, Technion – Israel Institute of Technology, Haifa 3200003, Israel.

^3^ The Department of Computer Science, University of California Los Angeles, Los Angeles 90095, CA, USA.

† To whom correspondence should be addressed:

Yonatan Belinkov, E-mail: [belinkov@technion.ac.il](mailto:belinkov@technion.ac.il)

Tal Pupko, E-mail: [talp@tauex.tau.ac.il](mailto:talp@tauex.tau.ac.il)

## Comparing different transformer architectures

We considered two different architectures for the transformers: “vaswani_wmt_en_de_big” (Vaswani et al., 2017) and “BART” (Lewis et al., 2020). Both types of transformers were trained applying the “*concat*” with the “*spaces*” representations. We tested the results on proteins datasets: PD1, PD2, PD3, and PD4. Of note, for the comparison to be fair, the two transformers were not pre-trained when applied to PD1 and thus their training started from random weights. Both architectures contain 16 attention heads, with an embedding size of 1,024, they differ in the details of their network design, including a different number of layers: 6 and 12 for “vaswani_wmt_en_de_big” and “BART”, respectively. The performance of these architectures was tested with several different sets of internal parameters (max tokens and learning rate). Both the coverage and the CS-score were higher for the “vaswani_wmt_en_de_big” architecture for the two datasets that are most difficult, i.e., PD3 and PD4 (Table S1). We thus selected this architecture for all analyses.

## Commands Used for Evaluating Aligners

We used the following commands to run the commonly used aligners:

### MUSCLE

muscle -in $INPUT_FILE -out $OUTPUT_FILE

### MAFFT

mafft --auto $INPUT_FILE > $OUTPUT_FILE

### PRANK

prank -d=$INPUT_FILE -o=$OUTPUT_FILE -f=8

ClustalW

clustalw2 -infile=$INPUT_FILE -outfile=$OUTPUT_FILE -output=FASTA

DIALIGN

dialign2-2 -fa -fn Dialign $INPUT_FILE

## Table S1

We trained two transformer architectures on the same amino-acid datasets and measured the alignment accuracy and coverage. The “*concat*” and “*spaces*” representations were used for input and output transformation, respectively. Datasets PD1, PD2, and PD3 are of pairwise alignments and dataset PD4 includes alignments of three sequences.

| PD1 | | | | | |
| --- | --- | --- | --- | --- | --- |
| Transformer name | Architecture | Max tokens | Learning rate | CS-score | Coverage |
| original | BART | 4096 | 5.00E-05 | 0.9996 | **0.9716** |
| alternative | BART | 2048 | 5.00E-05 | **0.9997** | 0.7724 |
| alternative_2 | BART | 4096 | 1.70E-05 | 0.9994 | 0.6161 |
| alternative_3 | BART | 2048 | 1.70E-05 | 0.9995 | 0.6039 |
| original | vaswani_wmt_en_de_big | 4096 | 5.00E-05 | 0.9995 | 0.9508 |
| alternative | vaswani_wmt_en_de_big | 2048 | 5.00E-05 | **0.9997** | 0.6882 |
| alternative_2 | vaswani_wmt_en_de_big | 4096 | 1.70E-05 | 0.9992 | 0.1616 |
| alternative_3 | vaswani_wmt_en_de_big | 2048 | 1.70E-05 | 0.9991 | 0.1436 |

| PD2 | | | | | |
| --- | --- | --- | --- | --- | --- |
| Transformer name | Architecture | Max tokens | Learning rate | CS-score | Coverage |
| original | BART | 4096 | 5.00E-05 | 0.9922 | 0.2279 |
| alternative | BART | 2048 | 5.00E-05 | **0.9983** | 0.8167 |
| original | vaswani_wmt_en_de_big | 4096 | 5.00E-05 | 0.997 | **0.8352** |
| alternative | vaswani_wmt_en_de_big | 2048 | 5.00E-05 | 0.9978 | 0.6348 |

| PD3 | | | | | |
| --- | --- | --- | --- | --- | --- |
| Transformer name | Architecture | Max tokens | Learning rate | CS-score | Coverage |
| original | BART | 4096 | 5.00E-05 | 0.7427 | 0.5976 |
| alternative | BART | 2048 | 5.00E-05 | 0.7644 | 0.5576 |
| original | vaswani_wmt_en_de_big | 4096 | 5.00E-05 | 0.8389 | **0.6464** |
| alternative | vaswani_wmt_en_de_big | 2048 | 5.00E-05 | **0.9362** | 0.511 |

| PD4 | | | | | |
| --- | --- | --- | --- | --- | --- |
| Transformer name | Architecture | Max tokens | Learning rate | CS-score | Coverage |
| original | BART | 4096 | 5.00E-05 | 0.9785 | 0.5054 |
| alternative | BART | 2048 | 5.00E-05 | 0.9878 | 0.8405 |
| original | vaswani_wmt_en_de_big | 4096 | 5.00E-05 | 0.993 | **0.995** |
| alternative | vaswani_wmt_en_de_big | 2048 | 5.00E-05 | **0.9949** | 0.9794 |

## Table S2

SpartaABC indel parameters as follow: for the rate of insertion ($R_{I}$), for the rate of deletion ($R_{D}$), a parameter for the insertion Zipfian distribution ($A_{I}$), a parameter for the deletion Zipfian distribution ($A_{D}$) and the root length. Which the latter is sampled uniformly from $(min range \times0.8, max range \times1.1)$. The order of the datasets refers to the order in which the transformers were trained. For example, the first nucleotide transformer was trained on dataset ND1, then the optimized weights were the starting point of ND2, etc. Tables (a), (b), (c) refer to nucleotide, protein datasets and a special table for specific datasets, respectively. “S” at the start of the dataset name, refers to a special dataset.

(a)

| Dataset name | Branch length | Root length | $\boldsymbol{R}_{\boldsymbol{I}}\boldsymbol{\&}\boldsymbol{R}_{\boldsymbol{D}}$ | $\boldsymbol{A}_{\boldsymbol{I}}\boldsymbol{\&}\boldsymbol{A}_{\boldsymbol{D}}$ | Number input sequences |
| --- | --- | --- | --- | --- | --- |
| ND1 | 0.03 - 0.1 | 50 - 60 | 0.0 - 0.05 | 1.01 - 2.0 | 2 |
| ND2 | 0.03 - 0.3 | 100 - 300 | 0.0 - 0.05 | 1.01 - 2.0 | 2 |
| ND3 | 0.3 - 0.6 | 200 - 300 | 0.04 - 0.05 | 1.01 - 2.0 | 2 |
| ND4 | 0.1 - 0.3 | 50 - 60 | 0.04 - 0.05 | 1.01 - 2.0 | 3 |
| ND5 | 0.15 | 55 | 0.5 | 1.0 - 1.01 | 3 |
| ND6 | 0.15 | 55 | 0.5 | 1.01 | 3 |
| ND7 | 0.15 | 55 | 0.5 | 1.5 | 3 |
| ND8 | 0.05 - 0.1 | 55 | 0.0 - 0.05 | 1.01 – 2 | 4 |
| ND9 | 0.05 - 0.1 | 55 | 0.03 - 0.05 | 1.01 – 2 | 4 |
| ND10 | 0.07 - 0.1 | 35 - 45 | 0.0 - 0.05 | 1.01 – 2 | 5 |
| ND11 | 0.08 - 0.09 | 37 - 42 | 0.03 - 0.05 | 1.01 – 2 | 5 |
| ND12 | 0.09 | 40 | 0.04 | 1.3 | 5 |
| ND13 | 0.05 - 0.1 | 55 | 0.02 - 0.03 | 1.0 - 1.1 | 4 |
| ND14 | 0.9 | 40 | 0.01 - 0.02 | 1.35 - 1.45 | 5 |
| ND15 | 0.07 - 0.1 | 35 - 45 | 0.0 - 0.05 | 1.01 – 2 | 7 |
| ND16 | 0.07 - 0.1 | 70 - 80 | 0.0 - 0.05 | 1.01 – 2 | 7 |

(b)

| Dataset name | Branch length | Root length | $\boldsymbol{R}_{\boldsymbol{I}}\boldsymbol{\&}\boldsymbol{R}_{\boldsymbol{D}}$ | $\boldsymbol{A}_{\boldsymbol{I}}\boldsymbol{\&}\boldsymbol{A}_{\boldsymbol{D}}$ | Number input sequences |
| --- | --- | --- | --- | --- | --- |
| PD1 | 0.03 - 0.05 | 30 - 40 | 0.0 - 0.05 | 1.01 - 2.0 | 2 |
| PD2 | 0.05 - 0.1 | 70 - 80 | 0.04 - 0.05 | 1.01 - 2.0 | 2 |
| PD3 | 0.1 - 0.3 | 200 - 250 | 0.04 - 0.05 | 1.01 - 2.0 | 2 |
| PD4 | 0.03 - 0.1 | 30 - 40 | 0.0 - 0.05 | 1.01 - 2.0 | 3 |
| PD5 | 0.1 - 0.2 | 50 - 60 | 0.04 - 0.05 | 1.01 - 2.0 | 3 |
| PD6 | 0.15 | 50 | 0.05 | 1.01 | 3 |
| PD7 | 0.15 | 50 | 0.05 | 1.5 | 3 |
| PD8 | 0.05 - 0.1 | 30 - 40 | 0.0 - 0.05 | 1.01 - 2 | 4 |
| PD9 | 0.075 | 35 | 0.03 | 1.07 | 4 |
| PD10 | 0.04 - 0.08 | 30 - 40 | 0.0 - 0.05 | 1.01 - 2 | 5 |
| PD11 | 0.04 - 0.08 | 30 - 40 | 0.0 - 0.05 | 1.01 - 2 | 6 |
| PD12 | 0.1 | 30 - 40 | 0.0 - 0.05 | 1.01 - 2 | 6 |
| PD13 | 0.1 | 30 - 40 | 0.03 - 0.05 | 1.01 - 2 | 6 |
| PD14 | 0.07 - 0.1 | 25 - 35 | 0.0 - 0.05 | 1.01 - 2 | 7 |
| PD15 | 0.08 - 0.09 | 27 - 32 | 0.03 - 0.05 | 1.01 - 2 | 7 |
| PD16 | 0.09 | 30 | 0.04 | 1.3 | 7 |
| PD17 | 0.05 - 0.1 | 40 | 0.0 - 0.05 | 1.01 - 2 | 10 |
| PD18 | 0.07 - 0.1 | 25 - 35 | 0.04 - 0.05 | 1.01 - 2 | 7 |

(c)

| Dataset name | Branch length | Root length | $\boldsymbol{R}_{\boldsymbol{I}}$ & $\boldsymbol{R}_{\boldsymbol{D}}$ | $\boldsymbol{A}_{\boldsymbol{I}}$ & $\boldsymbol{A}_{\boldsymbol{D}}$ | Number input sequences |
| --- | --- | --- | --- | --- | --- |
| SND1 | 0.05 - 0.1 | 40 | 0.0 - 0.05 | 1.01 - 2.0 | 10 |
| SPD1 | 0.05 - 0.1 | 40 | 0.0 - 0.05 | 1.01 - 2.0 | 10 |
| SPD2 | 0.05 - 0.1 | 40 | 0.0 - 0.05 | 1.01 - 2 | 10 |
| SPD3 | 0.07 - 0.1 | 25 - 35 | Dynamic | 7 |  |

## Fig. S1

The effect of indel parameters on BetaAlign performance: (a) The effect of $R_{I}$ and $R_{D}$ (in this case $A_{I}$ and $A_{D}$ were sampled from the entire range); (b) The effect of $A_{I}$ and $A_{D}$ (in this case $R_{I}$ and $R_{D}$ were sampled from the entire range). Figure illustrates the results on protein dataset SPD3. The red/blue colors represent the high/low regions, respectively (red regions are those that resulted in higher error rates). The shades are only to ease the visualization.


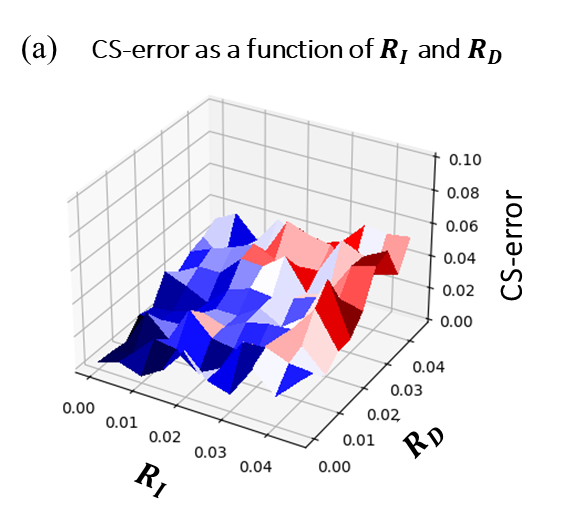

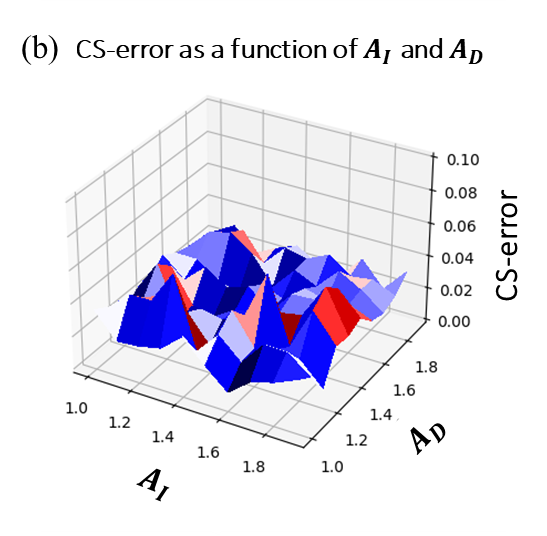


## Fig. S2

Example of compressing the embedding vector to a fixed size. Consider (a) to be the embedding of three sequences, two of length 4 nucleotides and one of length 5 nucleotides. The embedding dimension is of $1,024 \times15$ as there are 15 characters is the input sentence (13 nucleotides and 2 separation characters) and each character is encoded in a numeric vector of size 1,024. The compressed vector is (b) is of size $1 \times5,120$ as each one of the input sequences and the pipe sign corresponds to 1,024 entries in the compressed vector. The four first columns in this matrix are averaged and the resulting column vector represents a fixed size vector for the first sequence. This vector is transposed to form c1. The vector representing the pipe character remains the same, it is just transposed to form the vector p1. The next five columns are averaged and transposed to form c2, etc. One should emphasize that the pre-compressed representation already integrates information from all sequences due to the transformer self-attention mechanism, and consequently, the compressed representation also integrates information from all sequences.


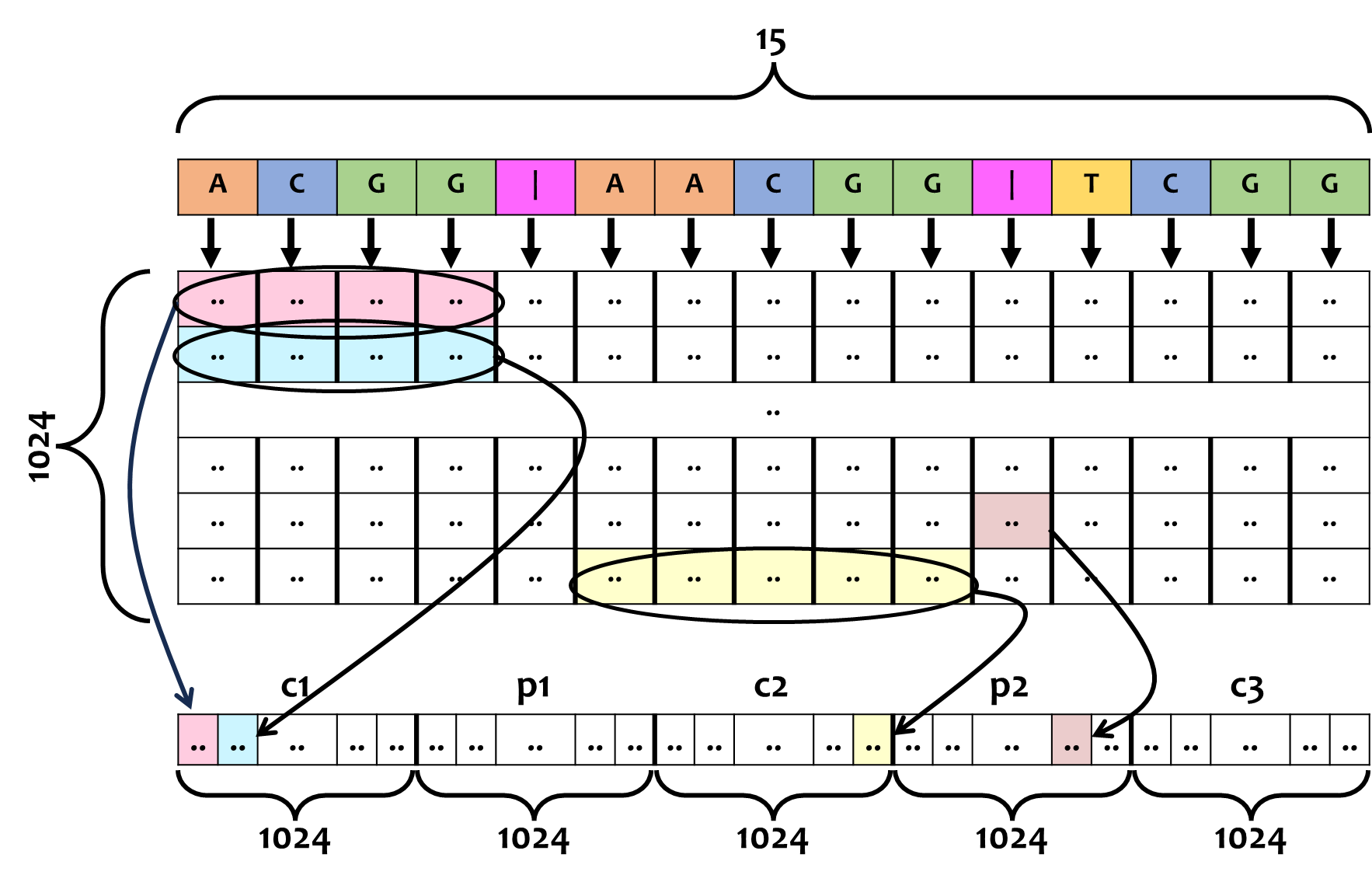


(a)

(b)
